# Low‐grade mixed neuroendocrine–non‐neuroendocrine neoplasm of the extrahepatic bile duct: A rare tumour with 11 years’ follow‐up before surgery

**DOI:** 10.1111/pin.13317

**Published:** 2023-03-07

**Authors:** Koji Kubota, Yoshinori Sato, Mai Iwaya, Tsuyoshi Notake, Akira Shimizu, Takuya Iguchi, Takeshi Uehara, Hiroyoshi Ota, Yuji Soejima

**Affiliations:** ^1^ Department of Surgery, Division of Gastroenterological, Hepato‐Biliary‐Pancreatic, Transplantation and Pediatric Surgery Shinshu University School of Medicine Matsumoto Japan; ^2^ Department of Laboratory Medicine Shinshu University Hospital Matsumoto Japan; ^3^ Department of Laboratory Medicine Shinshu University School of Medicine Matsumoto Japan; ^4^ Department of Clinical Laboratory Sciences, School of Health Sciences Shinshu University Matsumoto Japan

**Keywords:** bile duct, low grade, mixed neuroendocrine–non‐neuroendocrine neoplasm

**Abbreviations**
CECT: contrast‐enhanced CT
MiNEN: mixed neuroendocrine‐non‐neuroendocrine neoplasm
NEN: neuroendocrine neoplasm
NET: neuroendocrine tumour
SRS: somatostatin receptor scintigraphy

To the Editor,

Neuroendocrine neoplasms (NENs) in extrahepatic bile ducts are extremely rare, accounting for only 0.2%–2% of NENs.^1^ In 2010, the World Health Organization proposed the term “mixed adeno‐neuroendocrine carcinoma”. However, in 2019 the term mixed neuroendocrine–non‐neuroendocrine neoplasm (MiNEN) was substituted because non‐neuroendocrine components are not carcinomas only (i.e., adenomas) and also because neuroendocrine components include low‐grade neuroendocrine tumours (NETs). However, most lesions in the biliary system classified as MiNENs are composed of adenocarcinoma and neuroendocrine carcinoma.^2^ We here report a case of low‐grade MiNEN (composed of NET G1 and low‐grade adenocarcinoma) of the extrahepatic bile duct with 11 years of follow‐up before resection.

A 46‐year‐old Japanese woman was found to have a mass lesion in the extrahepatic bile duct during a routine health check. Abdominal contrast‐enhanced CT (CECT) revealed a 10 × 10 mm tumour in the distal bile duct (Supporting Information: Figure S1a, left panel). The lesion was well‐circumscribed and no biliary tract dilation nor enlarged lymph nodes were identified. Endoscopic retrograde cholangiography showed a smooth‐surfaced defect in the distal bile duct. A primary bile duct stromal tumour, possibly NEN, was suspected. Surgery was recommended; however, the patient was reluctant to agree to this and opted to attend for follow‐up, including periodic imaging, every 3–6 months. After slow growth of the lesion had been observed during 11 years of follow‐up, the patient finally agreed to undergo surgery. Abdominal CECT showed the same contrast pattern as seen on the initial examination. The tumour had increased in size to 18 × 14 mm and lymph nodes on the dorsal side of the portal vein had slowly enlarged during follow‐up; metastasis was therefore suspected (Supporting Information: Figure S1a, right panel). Fluorine‐18‐fluorodeoxyglucose positron emission tomography showed uptake consistent with the tumour and enlarged lymph nodes (Supporting Information: Figure S1b). A [111In‐DTPA‐D‐Phe1]‐octreotide scintigraphy, a form of somatostatin receptor scintigraphy (SRS), showed no uptake in the tumour (Supporting Information: Figure S1c). Biopsy of the bile duct showed NET with a low Ki‐67 labelling index. In light of the above preoperative clinicopathological findings, the patient underwent subtotal stomach‐preserving pancreatoduodenectomy when she was 56 year old. There has been no evidence of recurrence in the 25 months since her surgery.

Gross examination of the operative specimen showed a greyish‐white, smooth‐surfaced, 20 × 12 mm tumour in the distal bile duct (Figure 1a). On circumferential section, the tumour was white, the greatest diameter being 12 × 7 mm. Histologically, the tumour was well‐circumscribed and composed of neuroendocrine and ductular components. The neuroendocrine component showed trabecular and solid patterns with indolent morphology and was diffusely immunoreactive to INSM 1, chromogranin A, synaptophysin, and somatostatin, and negative for CK 7 (Figure 1b–e). No mitosis was identified and the Ki‐67 labelling index was less than 1%. On the basis of these findings, NET G1 was diagnosed. There was perineural and lymphovascular invasion in the NET component. The ductular component consisted of small, fairly uniform ducts. The cells forming the ducts were cuboidal with bland nuclei and abundant dense eosinophilic cytoplasm and were immunoreactive to CK 7, MUC 6, and CD 10 and negative for CDX 2, INSM 1, and synaptophysin. The Ki‐67 labelling index was less than 1%. There was no lymphovascular or perineural invasion by the ductular component. Some merging of the neuroendocrine and ductular portions of tumour was identified. The immunoprofiles of the ductular component and adjacent peribiliary ducts were the same however, the ductular component showed more enlarged nuclei and structural atypia than did the non‐neoplastic peribiliary ducts, which is in keeping with low‐grade adenocarcinoma (Supporting Information: Figure S2). No neuroendocrine cells were identified in the peribiliary ducts.The metastases in lymph nodes comprised only the NET component. The immunohistochemical profile is summarised in Supporting Information: Table S1.

**Figure 1:**
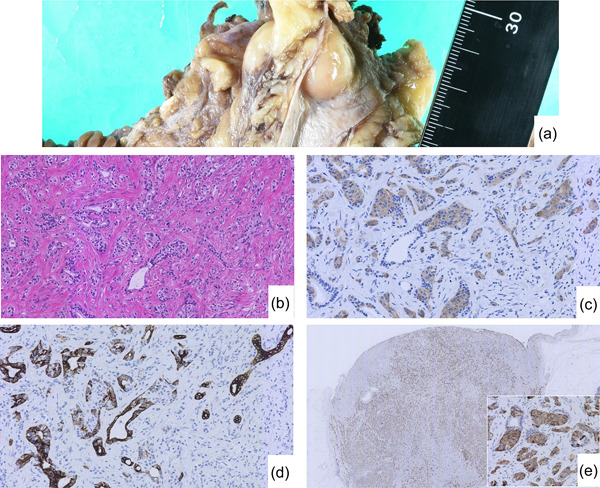
(a) Grossly, the tumour has a smooth surface. (b) The tumour is composed of ductular and neuroendocrine components. There is some merging of these components. (c) Synaptophysin is diffusely immunoreactive for the neuroendocrine component. (d) In contrast, CK7 is diffusely immunoreactive for the ductular component and negative for the neuroendocrine component. (e) The neuroendocrine component is diffusely immunoreactive for somatostatin.

We here report, to the best of our knowledge, the first published case of low‐grade MiNEN (composed of NET G1 and low‐grade adenocarcinoma) of the extrahepatic bile duct. This patient was followed up for 11 years before undergoing resection of the tumour. The ductular component had bland morphology; however, it showed more cytological atypia than the adjacent peribiliary ducts and there was some merging of the neuroendocrine and ductular portions of the tumour. It was therefore diagnosed as a MiNEN.

Not all MiNEN are carcinomas. They occasionally consist of a combination of two low‐grade components. Particularly in the lower GI tract, series of combined adenoma and low‐grade NET tumours have been reported and classified as composite intestinal adenoma–microcarcinoids.^3^ Similarly, in 2003, Deshpande et al.^4^ reported pancreatic NETs with neoductular differentiation; they named these as ductulo‐insular pancreatic endocrine neoplasms (DI‐PETs). The morphology of the ductular component was similar to that in our case: uniform small glands composed of eosinophilic cuboidal cells. Additionally, they described merging of the endocrine and ductular portions of the tumour and identified a ductulo‐insular unit with evidence of amphicrine differentiation by electron microscopy. In their study, 16.3% of pancreatic NETs were classified as DI‐PETs. These authors reported that the biologic behaviour of DI‐PETs is fundamentally the same as that of pancreatic NETs, the prognosis being better and similar to that of pancreatic NETs. As in our case, one of their patients had lymph node metastases in which only a NET component was identified.

Our patient's tumour had both NET G1 and bland neoductular differentiation. According to previous reports and the current World Health Organization classification, these findings are consistent with low‐grade MiNEN (NET G1 together with low‐grade adenocarcinoma with features of peribiliary ducts). However, caution is needed when diagnosing a MiNEN of the extrahepatic bile duct because most of these tumours are high grade (composed of adenocarcinoma and neuroendocrine carcinoma) and known to have a poor prognosis. A detailed justification for this diagnosis should therefore be provided by the pathologist.

Our 11 years of follow‐up data without any intervention is clinically valuable because it enhances our knowledge of the natural history of these tumours. During those 11 years, the tumour did not cause obstructive jaundice or any other detectable complications besides regional lymph node metastasis. Consecutive imaging measurements indicated a tumour doubling time of 4.65 years, highlighting how these tumours differ from typical MiNENs of the bile duct, the median overall survival of which is reportedly 12.2 months.^2^

SRS has been reported to assist diagnosis of gastroenteropancreatic NENs. The rates of positive SRS are 87% for Ki‐67 index ≤ 2%, 96% for Ki‐67 index 2%–15%, and 69% for Ki‐67 index > 15%. SRS is more helpful for lower grades of NEN.^5^ SRS is expected to be equally effective for primary, Grade 1 bile duct NENs. However, despite its NET component, our patient's tumour was immunoreactive for somatostatin, no uptake being observed. The specific explanations for this are not understood. Thus, additional cases are needed to evaluate the specificity and sensitivity of SRS for NETs arising in the bile duct.

In conclusion, we here report a case of low‐grade MiNEN composed of NET G1 and low‐grade adenocarcinoma with an 11‐year follow‐up before surgery. Our data support the indolent natural history of these tumours. The NET component was immunoreactive for somatostatin; however, this component was not detected by SRS. Further cases are needed to clarify the characteristics of these rare tumours.

## AUTHOR CONTRIBUTIONS

Each author has participated sufficiently in the work to take public responsibility for appropriate portions of the content: KK, TN, TI and AS collected clinical data. KK, YS and MI drafted the manuscript. MI, YS and TU analyzed histopathological features. YS and HO supervised the manuscript.

## CONFLICT OF INTEREST STATEMENT

The authors declare that they have no conflict of interest.

## DISCLOSURE

None declared.

## ETHICAL APPROVAL

The investigation was conducted in accordance with the Declaration of Helsinki of 1975. Our institution does not require an ethic approval for a case report.

## Supporting information

Table S1 Immunohistochemistry for tumor components and peribiliary duct.

Figure S1. Radiological findings of the tumor.

Figure S2. Histological findings of ductular component and peribiliary ducts.
