# Supplementary material for: Low‐grade mixed neuroendocrine–non‐neuroendocrine neoplasm of the extrahepatic bile duct: A rare tumour with 11 years’ follow‐up before surgery
Source: Pathol Int. 2023 Mar 7;73(4):173–6. doi: 10.1111/pin.13317 (PMC11551804; doi:10.1111/pin.13317)
Supplement: Supplementary file 1 — Table S1 Immunohistochemistry for tumor components and peribiliary duct. [file PIN-73-173-s003.docx]

Table S1 Immunohistochemistry for tumor components and peribiliary duct

| Antibody | NET | Ductular component | Peribiliary duct |
| --- | --- | --- | --- |
| INSM1 | +++ | - | - |
| Synaptophysin | +++ | - | - |
| Chromogranin A | +++ | - | - |
| Somatostatin | +++ | - | - |
| Gastrin | + | - | - |
| Serotonin | - | - | - |
| Insulin | - | - | - |
| CK7 | - | +++ | +++ |
| MUC6 | - | +++ | +++ |
| CD10 | + | +++ | ++ |
| P53 | wild | wild | wild |
| Ki67 (labeling index) | < 1% | < 1% | <1 % |
| IMP3 | - | - | - |
| CDX2 | - | - | - |
| CK20 | - | - | - |
