# Supplementary figures and images for: Low‐grade mixed neuroendocrine–non‐neuroendocrine neoplasm of the extrahepatic bile duct: A rare tumour with 11 years’ follow‐up before surgery
Source: Pathol Int. 2023 Mar 7;73(4):173–6. doi: 10.1111/pin.13317 (PMC11551804; doi:10.1111/pin.13317)

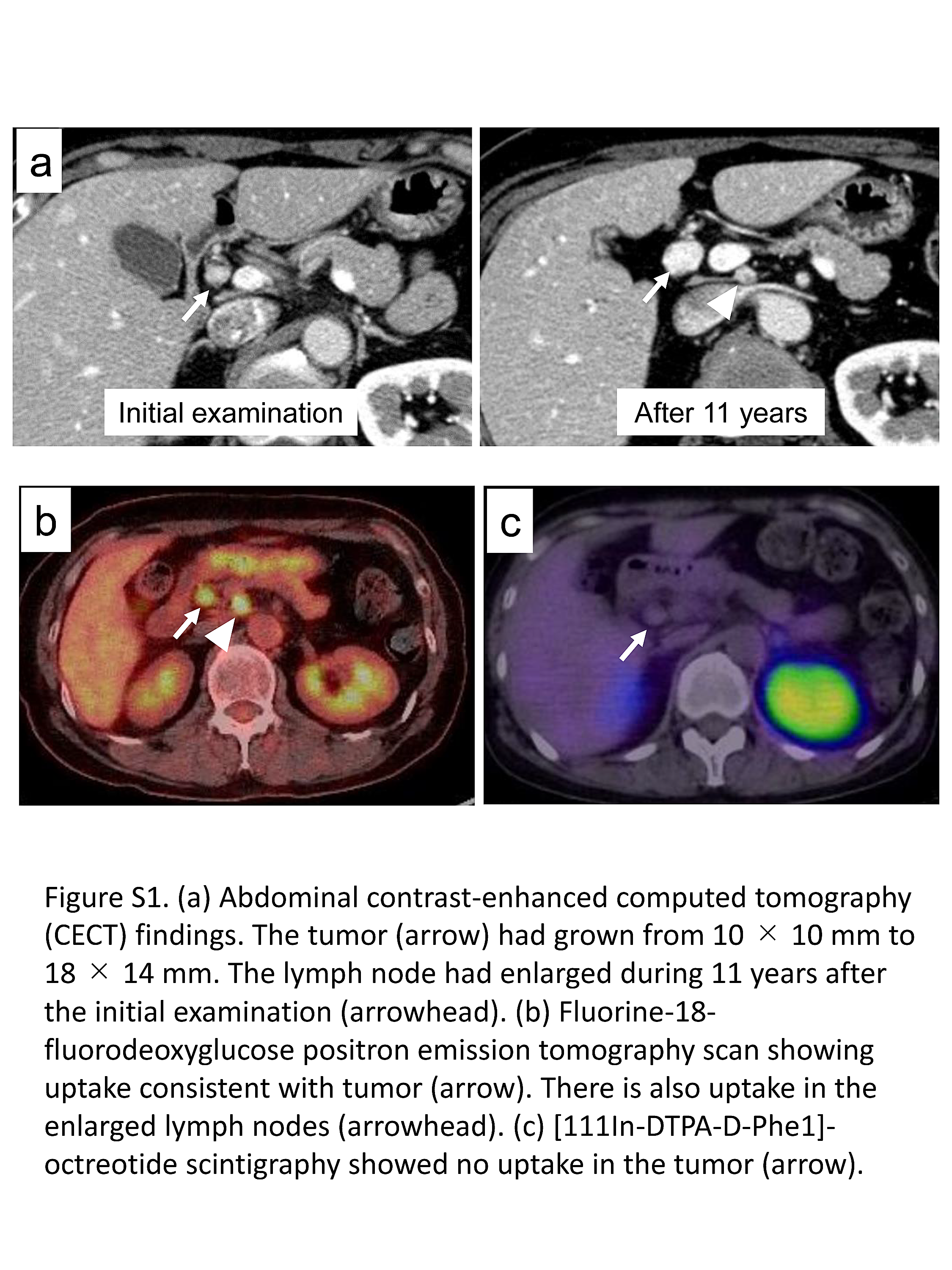

Supplement: Supplementary file 2 — Figure S1. Radiological findings of the tumor. [file PIN-73-173-s002.tif]

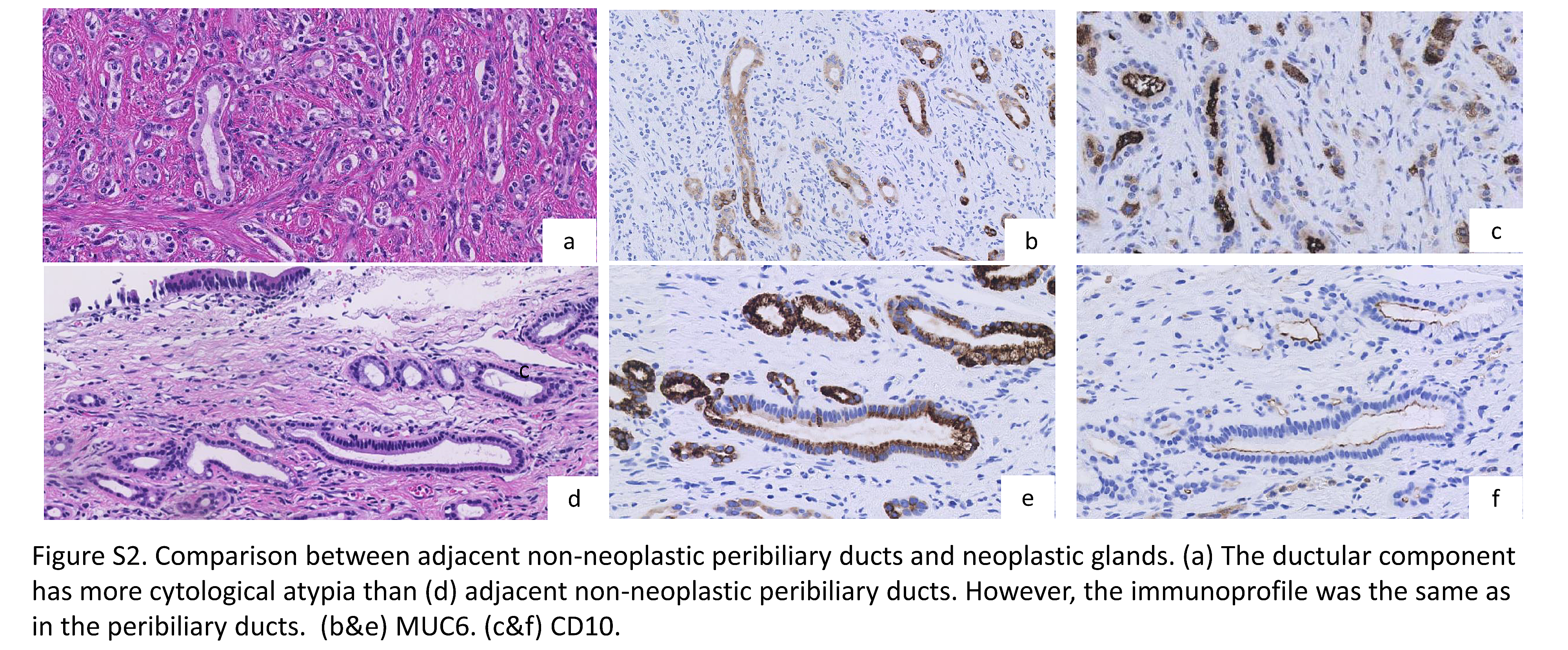

Supplement: Supplementary file 3 — Figure S2. Histological findings of ductular component and peribiliary ducts. [file PIN-73-173-s001.tif]
